# Operational Advantages of Novel Strategies Supported by Portability and Artificial Intelligence for Breast Cancer Screening in Low-Resource Rural Areas: Opportunities to Address Health Inequities and Vulnerability

**DOI:** 10.3390/medicina61020242

**Published:** 2025-01-30

**Authors:** Wolmark Xiques-Molina, Ivan David Lozada-Martinez, Ornella Fiorillo-Moreno, Angel Luis Hernández-Lastra, Valmore Bermúdez

**Affiliations:** 1 Research & Development Unit, Cure Latam Health Technologies, Barranquilla 080001, Colombia; dir.tecnico@curelatam.com; 2 Biomedical Scientometrics and Evidence-Based Research Unit, Department of Health Sciences, Universidad de la Costa, Barranquilla 080001, Colombia; 3 Clínica Iberoamérica, Barranquilla 080001, Colombia; ornella.fiorillo11@gmail.com; 4 Clínica El Carmen, Barranquilla 080001, Colombia; 5 Departamento de Hematooncología, Organización Clínica Bonnadona Prevenir, Barranquilla 080001, Colombia; angelhlastra@hotmail.com; 6 Centro de Investigaciones en Ciencias de la Vida, Facultad de Ciencias de la Salud, Universidad Simón Bolívar, Barranquilla 080001, Colombia

**Keywords:** breast neoplasms, mass screening, early detection of cancer, poverty areas, rural population, socio-economic factors

## Abstract

Early detection of breast cancer plays a crucial role in reducing the number of cases diagnosed at advanced stages, thereby lowering the high healthcare costs required to achieve disease-free survival and helping to prevent avoidable premature deaths. However, women living in rural and low-income areas face multiple obstacles that limit their access to conventional screening methods, such as mammography, which has been widely proven effective, particularly in high-income countries. The main barriers include a lack of healthcare infrastructure, long distances to medical facilities, high costs associated with large-scale screening programs, and shortages of specialized personnel. In this context, emerging technologies offer innovative solutions with the potential to mitigate these challenges. The development of strategies supported by artificial intelligence and the use of portable devices capable of overcoming geographical and sociocultural barriers represent valuable complementary tools for strengthening community-driven screening programs and expanding the reach of large-scale initiatives. However, to date, no comprehensive analysis has been conducted on the availability of evidence assessing the outcomes of breast cancer screening programs in vulnerable and underserved communities. This manuscript outlines the benefits of emerging portable technologies powered by artificial intelligence for detecting significant breast lesions in low-resource rural areas, where traditional screening methods are often difficult to implement. It also highlights gaps in the current knowledge, drawing on the available evidence. A search using PubMed yielded 7629 articles on breast cancer screening, of which only 59 (0.77%) addressed resource-limited settings and rural populations. Further filtering identified 29 original studies (0.38%) with specific epidemiological designs involving humans as the unit of analysis. The findings revealed significant disparities in evidence availability: nine studies originated from high-income countries, while fewer than half were from low-income or lower middle-income countries. Only two studies were conducted in Latin America, specifically in Peru and Argentina. This limited evidence poses challenges for generalizing and replicating recommendations for unexplored settings.

## 1. Introduction

Breast cancer screening is a key tool for reducing the number of cases diagnosed at advanced stages of the disease, lowering the high healthcare costs required to achieve disease-free survival and ultimately reducing potentially avoidable premature mortality [1]. However, there are significant disparities in the implementation of screening programs between rural and urban areas and between countries with different socio-economic levels [2]. Women in rural and low-income areas face numerous barriers that limit their access to traditional screening methods [3], such as mammography, which has proven to be effective, particularly in high-income countries [4]. Common barriers include a lack of healthcare infrastructure, long distances to medical facilities, high costs associated with large-scale screening initiatives, and staff shortages [5]. In contrast, medical services and access to advanced technologies in urban areas and more affluent countries allow for earlier detection and timely treatments [6]. As a result, the incidence of late-stage diagnosis is significantly higher in less advantaged regions, leading to poorer health outcomes [7].

This topic has been widely discussed by various international authors. They emphasize the importance of using evidence-based medicine and applying evidence in real-world decision-making. Key arguments, such as balancing benefits and risks, cost–utility, and cost–benefit analysis, are particularly important when addressing major public health challenges [8]. Thus, for populations facing considerable socio-economic and healthcare vulnerabilities, it is essential to implement strategies that are reproducible, scalable, and capable of being evaluated prospectively.

Given that traditional strategies such as mammography, ultrasound, or clinical breast exams require specific criteria to be operational [9], they are often unfeasible in many cases. New technologies offer innovative opportunities, with the potential to address this dilemma. Strategies and technologies supported by artificial intelligence (AI) and portable devices that overcome geographic and cultural barriers represent invaluable complementary tools for empowering community-driven programs and large-scale screening initiatives [10,11,12]. Breast cancer is one of the most common cancers worldwide [13]. However, despite widespread recognition of disparities in breast health, there has been little focus on the benefits and potential solutions offered by innovative screening methods for detecting clinically significant breast lesions in low-resource and rural settings. This manuscript outlines the benefits of emerging portable technologies powered by artificial intelligence in detecting significant breast lesions in low-resource rural areas, where traditional screening methods are often difficult to implement. It also highlights gaps in the current knowledge, drawing on the available evidence.

## 2. Inequalities in Access to Breast Cancer Screening and Their Impact on Population Health Outcomes

In low- and middle-income countries, such as those in Latin America, sub-Saharan Africa, and Asia, breast cancer screening rates are significantly low [3]. For instance, a study that assessed 15 low-income countries found that less than 5% of women aged 40 to 69 had undergone a mammogram in the past five years [3]. This fact contrasts sharply with high-income countries, where screening rates exceed 70% [4]. These disparities are largely driven by the lack of infrastructure and limited access to trained healthcare professionals, particularly in rural and resource-poor areas, where the scarcity of medical resources and challenging geography exacerbate the issue [5].

Economic barriers are widely recognized as one of the main factors limiting widespread breast cancer screening. The cost of screening tests and general healthcare is prohibitive for many women in low-income countries, particularly in rural areas where women often rely on overburdened public health systems with limited resources [14]. Moreover, disparities in educational levels and a lack of awareness regarding the importance of screening also play a crucial role in the low participation rates in early detection programs in these regions [15,16]. Another significant barrier is cultural perception and restriction. In Colombia, for example, a multi-ethnic and multicultural country, a notable percentage of indigenous communities and minorities restrict access to healthcare personnel due to concerns about the vulnerability of their traditions [15]. Consequently, traditional outreach strategies are not easily applicable in these cases, as additional limitations must be addressed at the local level through comprehensive strategies that directly involve community preferences, desires, and the acceptability of screening programs [17].

How do these barriers directly impact the population’s health outcomes and health development indicators? It has been observed that breast cancer mortality rates are significantly higher in low- and middle-income countries compared to high-income countries, despite the lower incidence in the former [13,18]. This phenomenon may occur because patients in less developed countries are often diagnosed with poorer prognoses at more advanced stages of the disease [15]. A study conducted using the U.S. National Cancer Database found that women living in rural and low-resource areas have a significantly higher likelihood of being diagnosed at an advanced stage of breast cancer, contributing to higher mortality rates [2].

Globally, in high-income countries like the United States, the five-year survival rate for breast cancer is approximately 90%. In contrast, in low- and middle-income countries, this figure can drop to as low as 40–50% [19]. The disparities in available resources, from healthcare infrastructure to early detection and treatment programs, largely explain these differences [5,6,7]. For this reason, recent clinical practice guidelines have raised questions about traditional strategies’ reproducibility and actual benefits for vulnerable and underserved communities [8]. Although these strategies have shown positive impacts over time, they have not been sufficient to meet the goals of reducing preventable early mortality or the healthcare costs associated with aggressive cancer treatments. In this context, priority should be given to innovative strategies that can effectively break down barriers to provide timely access to mass screening, with structured and timely referrals to specialized services and follow-up for appropriate treatment and rehabilitation [20]. These strategies must be implemented with the limited resources available to healthcare institutions and insurance providers in these settings.

## 3. Available Evidence on Breast Cancer Screening Outcomes in Vulnerable and Underserved Communities: A Gap Hindering Reproducible Evidence-Based Decision-Making

**Figure 1:**
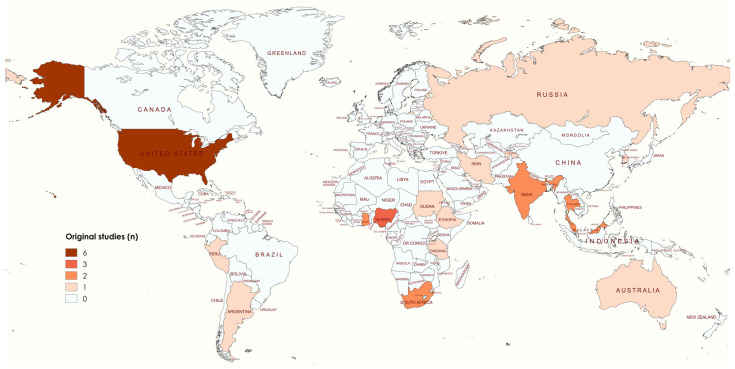
Countries with original studies published in PubMed related to breast cancer screening in vulnerable or poor rural communities.

To explore the knowledge gap and the available evidence regarding the characteristics, experiences, and outcomes related to breast cancer screening in vulnerable communities and resource-limited settings, including rural populations, a brief scientometric analysis was conducted using the PubMed search engine on 9 October 2024. The search utilized MeSH terms along with synonyms for “Breast Neoplasms”, “Mass Screenings”, “Resource-Limited Settings”, and “Rural Population”. These terms were combined with specific tags for “Title” and “Title/Abstract”. A total of 7629 results were found when combining terms related to any breast cancer screening (See Supplementary Materials for details). However, when limiting the search to terms associated with resource-limited settings and rural populations, only 59 results were retrieved, representing 0.77% of the available evidence. After further filtering for original studies (studies with specific epidemiological designs and humans as the unit of analysis), only 29 articles were identified (0.38%; *n* = 29/7629) (Figure 1).

A significant disparity in the availability of evidence was identified, as nine articles originated from high-income countries, while seven came from seven middle- and high-income countries. These studies primarily focused on the performance of screening programs in rural areas and settings with geographic and sociodemographic barriers. However, this reveals that just under 50% of the evidence comes from low- or lower middle-income countries, posing a substantial challenge to the generalization and reproducibility of recommendations and strategies in unexplored settings. Specific outcomes supporting screening tools or describing barriers that should be considered in decision-making remain largely unknown in these areas. Only two studies from Latin America were identified, specifically from Peru [21] and Argentina [22]. An important gap in the evidence has recently been described in Latin America, which confirms this finding [23]. Yet, with their diverse cultural, social, economic, and healthcare landscapes, other countries in the region require the necessary data to assess the feasibility of designing community-based primary care programs tailored to population characteristics and specific barriers. Based on these findings, it can be inferred that many recommendations for the use of traditional strategies—such as mammography, ultrasound, or clinical breast exams—issued by various scientific societies or organizations lack the rigorous, high-quality evidence needed to support their use, validity, and reproducibility in real-world settings.

Based on scientific principles like the Leiden Manifesto and evidence-based research approaches [24], evidence-based decision-making requires a foundation of rigor, transparency, relevance, reproducibility, and precision. This process relies on locally relevant research that evaluates mixed health measures, considers the research context and the perspectives of information end-users, and clearly defines the real-world implications of the findings [24]. Given that the current evidence on breast cancer screening in resource-limited and rural settings is notably scarce, strategies must be designed with flexibility and attention to the specific needs of each region. These strategies should balance the benefit–risk of interventions, ultimately aiding in the detection of clinically relevant breast lesions while adhering to the comprehensive healthcare and breast cancer prevention pathways of each country. Furthermore, these findings highlight the need to design and implement studies that specifically assess perceptions, accessibility, timeliness, preferences, and quantitative outcomes regarding the effectiveness of community-based screening strategies for early breast cancer detection. Such research should also identify potential tools to help overcome geographic, social, cultural, economic, and healthcare barriers in rural and resource-limited areas.

## 4. Portable Devices and the Use of AI to Implement Community-Based Primary Care Programs: An Emerging Opportunity

In light of the barriers described to the implementation of community-based breast cancer screening programs in low-resource settings and rural areas, certain devices and innovative strategies show promise in significantly addressing the issue. AI in community-based primary care offers various multilevel advantages, ranging from resource optimization to improvements in clinical outcomes and access to healthcare services [25,26]. These tools make it possible to develop personalized predictive models, AI-assisted diagnostic systems, telemedicine and remote monitoring, reduction in human bias, systematic detection of epidemiological patterns, and identification of social factors associated with the effectiveness or failure of screening cycles [27,28]. The World Economic Forum [29] and the Food and Drug Administration (FDA) [11] have highlighted the emergence of technology-assisted solutions being integrated into health systems, where monitoring, follow-up, and information flow enhance surveillance and timely treatment opportunities, providing a favorable cost–benefit and cost–utility balance compared to traditional strategies [29]. These conventional approaches lack specific indicators within comprehensive care pathways and rely heavily on human and logistical management.

Portable devices represent an innovative solution to geographic barriers, high-cost infrastructure, the need for highly specialized human talent, and social investment in human-operated information systems [10,12]. Increasingly, battery-powered devices, primarily based on ultrasound [10] or elastography [12], are being used to detect clinically relevant breast lesions by identifying abnormalities in breast tissue. Unlike portable mammography, which also requires specific operational criteria and whose performance can vary compared to traditional mammography [30], these newer devices offer comparable performance. Coupled with AI, featuring automatic reading and calibration as well as machine learning, current portable devices have the potential to improve their performance over time [12]. They could even be operated by community volunteers in rural areas, traditional ethnic communities, and populations facing significant inequities in accessing timely screening [12]. Some of these devices, which have shown acceptable performance as screening tools, are designed with a primary focus on high sensitivity to detect the maximum number of suspicious cases, which can then be evaluated with a higher-performance diagnostic tool [31].

**Figure 2:**
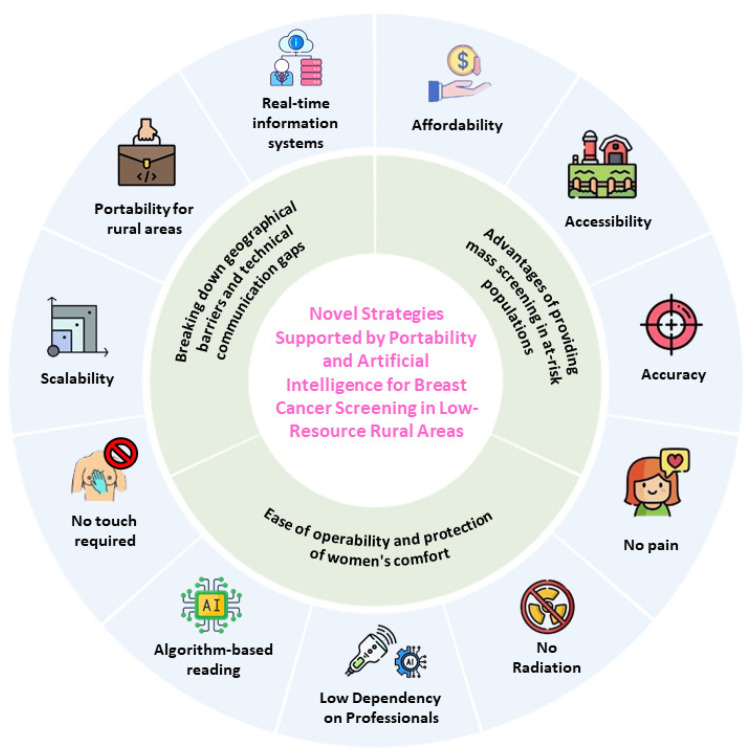
Advantages of using portable devices and artificial intelligence for developing novel community-based strategies for breast cancer screening in low-resource settings. Source: authors.

These low-cost devices, which have a shorter learning curve and greater operational efficiency [32], present an opportunity to promote their manufacturing and validation in low- and middle-income countries. This would enable their adaptation to each region’s breast cancer screening pathways, while supporting the development of personalized, community-based primary care strategies. Unlike mammography, which can cause side effects such as pain or low-level radiation exposure [33], these innovative portable devices have demonstrated high levels of patient satisfaction, encouraging strong adherence to repeated screenings over time [12] (Figure 2).

AI-based devices, such as thermal imaging combined with AI algorithms, can be used without extensive infrastructure, facilitating the implementation of mass screening programs [34]. The precision and efficiency of these new devices can enhance diagnostic accuracy compared to traditional techniques like clinical breast exams, which may struggle to detect small lesions in dense breast tissue [35]. Palpation during clinical breast exams often causes discomfort due to the exposure and manipulation of the breasts [36]. Portable devices help mitigate this discomfort. With machine learning and AI-supported algorithms, there is a low dependency on professional skills when using these new portable devices [10,12] compared to clinical breast exams and even mammography, which requires a high level of operator concordance—something that is weak in various studies [37]. Finally, AI integration can be incorporated into mobile systems, enabling remote assessments and diagnoses while maintaining diagnostic performance as a screening tool [38]. This ensures favorable scalability in remote areas during early breast cancer detection programs.

Lastly, the current limitations of integrating AI-based tools into healthcare delivery must be carefully considered. From a bioethical perspective, the implementation of AI tools in vulnerable communities must ensure equitable access and prevent any form of implicit or explicit discrimination. It is essential to respect cultural traditions and local concerns to foster community acceptance. Furthermore, ensuring data privacy and security is critical, as the use of AI involves the collection of sensitive information. Communities must be clearly informed about how their data will be managed and must provide consent in a transparent and comprehensible manner.

From a logistical perspective, the infrastructure in resource-limited areas presents a significant challenge. Many communities lack reliable access to electricity or connectivity, limiting the functionality of AI devices that depend on digital networks. The cost of acquiring, maintaining, and repairing these tools can also be a considerable barrier. Strategic planning is needed to ensure that these technologies are sustainable over time. Additionally, local healthcare workers must be trained to operate and supervise these devices, with training programs tailored to basic education levels.

## 5. Conclusions

Given the current barriers faced by breast cancer screening in increasing the timely detection of clinically relevant breast lesions and reducing the number of late-stage diagnoses in low-resource rural areas, it is essential to consider the operational advantages of innovative strategies supported by complementary portable devices and AI. These technologies can improve accessibility and provide timely access to effective screening services, addressing vulnerability and inequity in healthcare when implementing primary or secondary breast cancer care. There is a significant gap in the knowledge and available evidence regarding the exploration of various factors that could strengthen the implementation of these emerging health technologies for mass breast cancer screening in low- and middle-income countries.

## Data Availability

Not applicable.

## Supplementary Materials

The following supporting information can be downloaded at: https://www.mdpi.com/article/10.3390/medicina61020242/s1, Supplementary File S1.
