# Supplementary material for: Operational Advantages of Novel Strategies Supported by Portability and Artificial Intelligence for Breast Cancer Screening in Low-Resource Rural Areas: Opportunities to Address Health Inequities and Vulnerability
Source: Medicina (Kaunas). 2025 Jan 30;61(2):242. doi: 10.3390/medicina61020242 (PMC11857370; doi:10.3390/medicina61020242)
Supplement: Supplementary file 1 [file medicina-61-00242-s001.zip › medicina-3399817-supplementary.pdf]

Supplementary File S1

PubMed search strategy

Date of search: 09/10/2024

#1

Breast Neoplasm

Breast Tumor

Breast Cancer

Cancer of Breast

Cancer of the Breast

Malignant Neoplasm of Breast

Breast Malignant Neoplasm

Malignant Tumor of Breast

Breast Malignant Tumor

Mammary Cancer

Human Mammary Neoplasm

Breast Carcinoma

Human Mammary Carcinoma

Inflammatory Breast Neoplasms

Triple Negative Breast Neoplasms

Unilateral Breast Neoplasms

Breast Ductal Carcinoma

Breast Lobular Carcinoma

((((((((((((((((Breast Neoplasm[Title]) OR (Breast Tumor[Title])) OR (Breast Cancer[Title])) OR (Cancer of Breast[Title])) OR (Cancer of the Breast[Title])) OR (Malignant Neoplasm of Breast[Title])) OR (Breast Malignant Neoplasm[Title])) OR (Malignant Tumor of Breast[Title])) OR (Breast Malignant Tumor[Title])) OR (Mammary Cancer[Title])) OR (Human Mammary Neoplasm[Title])) OR (Breast

Carcinoma[Title])) OR (Human Mammary Carcinoma[Title])) OR (Inflammatory Breast Neoplasms[Title])) OR (Triple Negative Breast Neoplasms[Title])) OR (Unilateral Breast Neoplasms[Title])) OR (Breast Ductal Carcinoma[Title])) OR (Breast Lobular Carcinoma[Title]) = 248,349 (9/10/2024)

#2

Mass Screenings

Screening

Early Detection of Cancer

Cancer Early Detection

Early Diagnosis of Cancer

Cancer Early Diagnosis

Cancer Screening

Cancer Screening Test

(((((((Mass Screenings[Title]) OR (Screening[Title])) OR (Early Detection of Cancer[Title])) OR (Cancer Early Detection[Title])) OR (Early Diagnosis of Cancer[Title])) OR (Cancer Early Diagnosis[Title])) OR (Cancer Screening[Title])) OR (Cancer Screening Test[Title]) AND ((((((((((((((((Breast Neoplasm[Title]) OR (Breast Tumor[Title])) OR (Breast Cancer[Title])) OR (Cancer of Breast[Title])) OR (Cancer of the Breast[Title])) OR (Malignant Neoplasm of Breast[Title])) OR (Breast Malignant Neoplasm[Title])) OR (Malignant Tumor of Breast[Title])) OR (Breast Malignant Tumor[Title])) OR (Mammary Cancer[Title])) OR (Human Mammary Neoplasm[Title])) OR (Breast Carcinoma[Title])) OR (Human Mammary Carcinoma[Title])) OR (Inflammatory Breast Neoplasms[Title])) OR (Triple Negative Breast Neoplasms[Title])) OR (Unilateral Breast Neoplasms[Title])) OR (Breast Ductal Carcinoma[Title])) OR (Breast Lobular Carcinoma[Title]) = 7629 (09/10/2024)

#3

Resource-Limited Settings

Austere Condition

Austere Environment

Resource Limited Area

Resource Limited Region

Low Resource Setting

Rural Population

Rural Spatial Distribution

Rural Communities

Rural Community

Rural Residence

Rural Residences

Rural Health Service

Rural Health Center

((((((((((((Resource-Limited Settings[Title/Abstract]) OR (Austere Condition[Title/Abstract])) OR (Austere Environment[Title/Abstract])) OR (Resource Limited Area[Title/Abstract])) OR (Resource Limited Region[Title/Abstract])) OR (Low Resource Setting[Title/Abstract])) OR (Rural Population[Title/Abstract])) OR (Rural Spatial Distribution[Title/Abstract])) OR (Rural Communities[Title/Abstract])) OR (Rural Community[Title/Abstract])) OR (Rural Residence[Title/Abstract])) OR (Rural Residences[Title/Abstract])) OR (Rural Health Service[Title/Abstract])) OR (Rural Health Center[Title/Abstract])

Final search

#1 AND #2 AND #3 = 28 results (09/10/2024)

#1 AND #2 (TIAB) AND #3 (TIAB) = 59 results (09/10/2024)

Countries with original studies according to the results

Country	Frequency
South Africa	2
Iran	1
Ethiopia	1
Tanzania	1
Nigeria	3
India	2
Thailand	2
United States	6
Peru	1
Russian Federation	1
Ghana	2
Malaysia	2
Australia	1
Tajikistan	1
South Korea	1
Sudan	1
Argentina	1
